# Pathways, technology and the patient—connected health through the lifecycle

**DOI:** 10.3389/fdgth.2023.1057518

**Published:** 2023-10-19

**Authors:** Silvana Togneri MacMahon, Ita Richardson

**Affiliations:** ^1^ Lero – the Science Foundation Ireland Research Centre for Software, Limerick, Ireland; ^2^ School of Computing, Dublin City University, Dublin, Ireland; ^3^ Department of Computer Science and Information Systems, University of Limerick, Limerick, Ireland

**Keywords:** connected health (CH), health information technology systems, healthcare pathways, standards, patient centred care

## Abstract

Connected Health solutions are ubiquitous in providing patient centered care and in responding to a new paradigm of care pathways where Health Information Technology is being introduced. This paper defines Connected Health, and, in particular, describes standards and regulations which are important to the implementation of safe, effective and secure Connected Health solutions. This paper provides: a holistic view of Connected Health; provides a standards and regulations based view of the lifecycle of the Health IT system; and identifies the relevant roles and responsibilities at the various stages of the lifecycle for both manufacturers of connected health solution and healthcare delivery organization solutions. We discuss how the implementation of standards and regulations, while implementing and using Health IT infrastructure, requires close collaboration and ongoing communication between Healthcare Delivery Organizations and Accountable Manufacturers throughout the lifecycle of the health IT system. Furthermore, bringing technology into the healthcare system requires a robust and comprehensive approach to Clinical Change Management to support the business and clinical changes that the implementation of such solutions requires. Ultimately, to implement safe, effective, and secure Connected Health solutions in the healthcare ecosystem, it requires that all those involved work together so that the main requirement—patient-centered care—is realized.

## 1. Introduction

Technology, both hardware and software, impacts every facet of our lives. It has become the norm in many of our everyday environments to use smart phones, internet, mobile technology, integrated software systems and ubiquitous computing. We increasingly shop and pay, make travel arrangements, access education, collaborate and socialize online—our day-to-day processes are being digitally transformed. We also note that, as the technology that supports them evolves, the processes through which these activities are undertaken must evolve. This can happen in either an *ad hoc* manner, where people “figure out” the processes as they use the technology, or in a managed manner, where people know and understand what they are doing. However, in order for them to be efficient and effective, the implementation of digital systems must be managed (1).

Healthcare is also experiencing digital implementation. This is taking place not only in the traditional healthcare setting of the hospital, clinics and the General Practitioner's office but, increasingly, it is taking place in the community. In addition, community healthcare has changed, and now focuses on wellbeing and prevention in the home. Technology is being used by those who are well and those who are ill, it is being used by individuals themselves and by their carers, and it is being used by family members and professional medical personnel.

**Figure 1:**
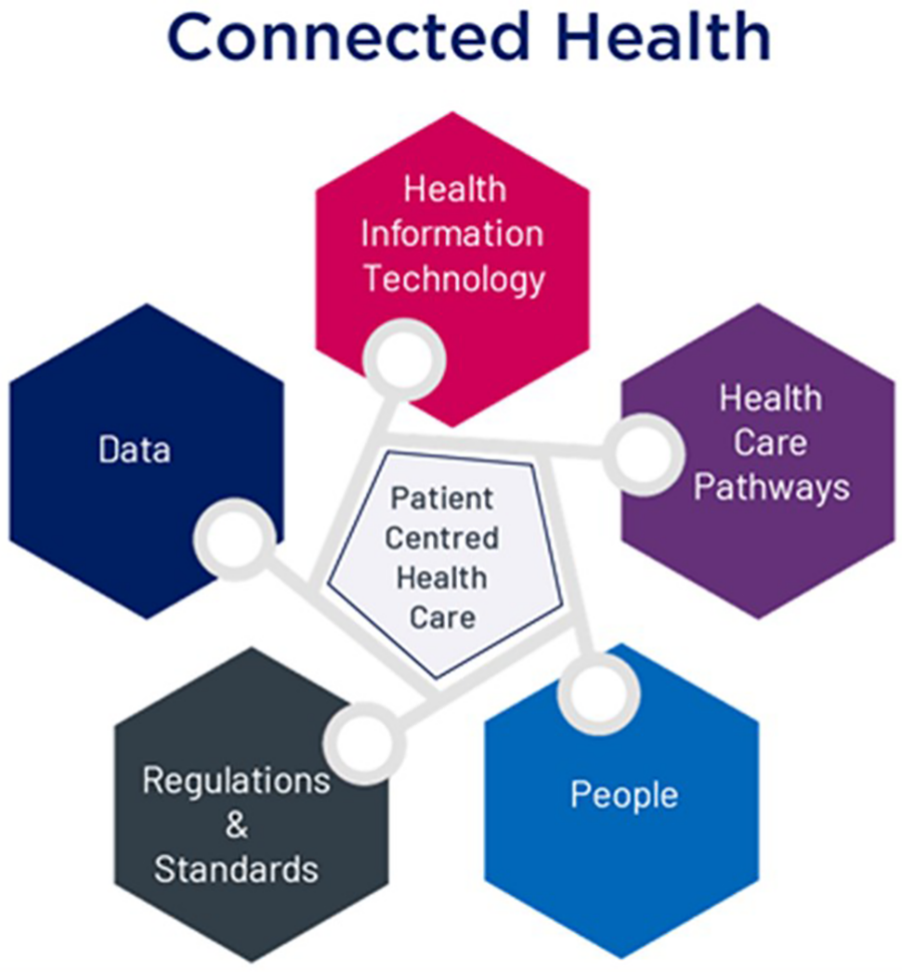
Connected health components provide patient-centered health care.

Health professionals are making increasing use of technology to monitor, diagnose, prescribe, maintain patient records, and generally enhance their healthcare practice. However, introducing health information technology to healthcare, commonly known as Connected Health, is broader than just “putting” hardware and/or software in place. Through our previous ongoing research on this topic (2), we have defined it as, and illustrated it in Figure 1:

“Connected Health is where patient-centered care results from following defined healthcare pathways undertaken by healthcare professionals, patients and/or carers who are supported by the use of health information technology (software and/or hardware), regulated when used as a Medical Device^1^, and facilitating appropriate health data sharing”.

The implementation of connected health systems has resulted in moving away from traditional interaction with healthcare systems. When the majority of healthcare systems were established, patients would attend care facilities for episodes of acute care. But, from a cost perspective and due to the lack of qualified personnel, combined with the increasing ageing population and a consequent prevalence of chronic disease, the way that care is provided has had to change (3). To manage chronic disease, we interact with our caregivers much more regularly and over a longer period of time. The definition of caregiver now includes family members and a broader range of healthcare professionals, and we see an increased emphasis on wellbeing and self-monitoring as a means to prevent and manage chronic disease. Technology is important in supporting these ongoing relationships (4). Care pathways are being altered so that sections of the pathways are being replaced by a connected health solution. For example, surgeons are now using robots to do the surgery, while they are driving the operation through computerization. While the move to connected health solutions is being driven by such changes to care pathways, it must also be recognised that the implementation of connected health solutions can in themselves require the modification and redesign of care pathways.

While recognizing the potential benefits of health information technology (IT), the patient will continue to be the most important consideration in the medical domain. Technology-supported healthcare pathways must be designed so that they result in quality outcomes for the patient, and to do this, patient-centered care must be provided. There is also increasing pressure to design healthcare pathways that provide for the efficient delivery of care to the patient with a focus on providing this care at a reduced cost. This combination of the traditional with the technological pathway requires process-driven health care delivery, so that each person within the pathway understands the role of people and the role of technology within that pathway. Parts of these pathways may require a healthcare professional, e.g., where medication is being prescribed, but parts of the pathway can be carried out independently, e.g., in instance where a decision support system is being used and the knowledge of the professional has been included in the system.

The use of connected health systems results in the development of increasingly complex health systems which includes health IT systems. These systems can be composed of regulated and unregulated technologies and devices, and components can be added and removed from the system throughout a long lifecycle. As the patient is the central focus of these systems, there is a need to ensure that, during and after the implementation of these connected health systems, they allow for patient-centered care. In this paper, we are particularly interested in the aspects of safety, effectiveness and security, and the standards and regulations which have been developed to support the maintenance of these properties through the lifecycle. Furthermore, the ongoing development and implementation of these standards and regulations requires close and continuous collaboration among health system stakeholders. Therefore, our Research Objective for this paper is to present the standards and regulations required to implement health IT systems so that they maintain the properties of safety, effectiveness and security.

In the remainder of the paper, Section 2 examines how the use of connected health has impacted the design and use of patient-centered care pathways. Section 3 examines the standards that must implemented be in order to protect safety, effectiveness and security of the Health IT Infrastructure and examines how these standards relate to the regulations that make up part of the Broader Healthcare System. Section 4 examines how the implementation of connected health solutions is managed through a process of Clinical Change Management within the Healthcare Delivery Context. Section 5 provides discussion and concludes that standards and regulations are vital for the implementation of connected health solutions, but that the implementation of such standards through the lifecycle must be completed as part of a comprehensive clinical risk management process to ensure the safety, effectiveness and security of health IT systems.

## 2. Connected health changing healthcare pathways

The management of chronic disease requires the establishment of an ongoing relationship between the patient and their care team, but recently, due to the recent downturn in the global economy, there is an increased focus on ensuring that a high standard of care is provided to the patient while reducing the cost of care (5). Furthermore, the patient-care team relationship requires carefully designed care processes which require the support of information technology (3, 5–7). Interoperable^2^ medical devices have been identified as one potential approach to achieving this goal (8–10). Recognising this potential, governments have provided incentives to promote the meaningful use of interoperable medical devices and health IT, such as Electronic Health Records (EHRs) (11–13). Consequently, the number of networked medical devices in use continues to increase (14–16).

The global Covid-19 pandemic has also increased the pace of use of connected health in healthcare, medical affairs and the pharmaceutical industry, with many countries adopting “digital first” strategies to control the spread of the virus and changing the way in which healthcare systems function at a pace not experienced before (17–19). The pandemic also highlighted the role of connected health systems in facilitating transparent data sharing (20). This rapid shift has not only served as a test for the maturity of health IT—it has also shifted patient's expectations about the way in which care is provided (21).

In order to address the diverse and complex challenges faced by global health services, a connected health ecosystem is required. The implications of such a system do not stop at the health IT systems level (22). Rather, the importance of considering the larger socio-technical environment is recognised. Each facet of the ecosystem and their interactions need to be implemented with a view to the relevant Standards and Regulations. This ecosystem includes: the Health IT Infrastructure; the Healthcare Delivery Context and the Broader Healthcare System used. The health IT infrastructure consists of the infrastructure itself (for example, hardware, software, networks, interfaces to other systems, medical devices and data), and the organizations involved in developing, implementing and operating the systems' components and services. The healthcare delivery context refers to the specific organization setting where the health IT system is being deployed and to the stakeholders of that system. Finally, the broader healthcare system encompasses for example, standards, regulations, funding and policy implications, within which the Healthcare Delivery Organization must comply and operate. It should be noted that this ecosystem exists within a larger external environment which can also have an impact. Examples include the recent global pandemic and, on a more general level, factors such as public opinion can also affect the ecosystem. However, our interest in this paper is specifically on presenting standards and regulations which are relevant to connected health systems. Due to the complexity of the systems and the various standards required for different safety classifications of devices, our intent here is not to provide an exhaustive list, but rather to highlight some areas for consideration during the connected health life cycle.

## 3. Standards and regulations

### 3.1. Standards—the *health it infrastructure* lifecycle

Health IT is defined as:

“Health information technology is the hardware, software, and systems that comprise the input, transmission, use, extraction, and analysis of information in the healthcare sector. The end-users of this technology include not only patients, physicians, and other front-line healthcare providers, but also medical researchers, healthcare insurance companies, public health agencies, regulatory and quality assurance entities, pharmaceutical and medical device corporations, and various levels of government” (23).

The need to support an ongoing relationship between the patient and the care giver has contributed not only the complexity of health IT systems but has also extended the lifecycle of these systems. These systems provide advanced levels of decision support and integrate patient data between systems, across health delivery contexts, and throughout the relationship between patient and caregiver (22). While these systems provide benefits to both the patent and to the wider healthcare system (24, 25), with the increasingly complex systems composed of regulated and unregulated technologies, there is also increased likelihood of software-induced adverse events (26–28). From a technical perspective, issues have been reported related to design flaws, coding errors, incorrect implementation or configuration, data integrity issues and faults in decision support tools. Poor alignment with clinical workflows and improper maintenance and use of such systems have also been reported and are provided as examples of events with the potential to cause harm to patients (22).

In addition, health IT systems need to support the three key properties of safety, effectiveness and security during this complex and extended lifecycle. Safety is defined as “freedom from unacceptable risk” (29). Effectiveness means that the system being used can produce the intended result for the patient and for the Organization operating the health IT system (30). In this context, security is considered from the perspective of both data and system security. This requires that, throughout the lifecycle, the system maintains an operational state in which information assets are reasonably protected from degradation of confidentiality, integrity, and availability (30). A number of standards that aim to support the safe development and operation of medical devices and health IT systems and regulations in the context of the broader healthcare system are discussed in this section.

Our particular interest is in standards (a) related to the design and development of medical device software and (b) those for the implementation and clinical use of these devices within a health IT system. In terms of regulation, we examine those within the European Union (EU), which is similar to other countries such as the U.S.A. Prior to examining the standards and regulations, the remainder of this section examines the composition of health IT Systems and their lifecycle.

A recently published standard, *ISO 81001-1:2021 Health software and health IT systems safety, effectiveness and security—Part 1: Principles and concepts* recognizes a need for a “common understanding of the relevant concepts, principles and terminology is important in standardizing the processes and inter-organizational communications to support a coordinated approach to managing safety, effectiveness and security” (22) and seeks to provide a framework to address these issues. As part of this framework, the lifecycle of health software and systems is defined. It should be noted that the health IT infrastructure itself, as well as each IT health component brought onto it, is following its own life cycle. In addition to defining the lifecycle, the standard recognizes a number of roles that share responsibility across the lifecycle as follows:

• Top Management—a group of people who direct, control and have overall accountability in a healthcare organization.
• System owner—accountable for ensuring the health software and health IT system being acquired and implemented will meet their organization's healthcare delivery services needs for its intended use.
• Developer—responsible for execution of the design and development phase (from concept through to release and maintenance) of a health IT system. A developer could be part of a manufacturer organization, a supplier of services or a Health Delivery Organization.
• Integrator—responsible for the technical installation, configuration, data migration and integration with the other health IT systems, medical devices and technology being used by the healthcare organization.
• Implementer—responsible for the clinical installation, workflow optimization and training in the clinical setting (an implementer may be the developer or owner).
• Administrator—responsible for the ongoing operation of the implemented health IT system and ensuring it is safeguarded and maintained on an ongoing basis to meet its design requirements.
• Users—Persons using the system in the clinical setting, which may include, for example, consumers in the case of personal health records.

The Health IT lifecycle consists of: Design and Development; Acquisition; Installation and Integration; Implementation; Operational use in the Clinical Context; and Decommissioning. The standard notes the importance of communication across the lifecycle stages and that communication across transition points in the life cycle is key to the maintenance of the key properties of the system. In complex systems with diverse stakeholders, communication is especially important and requires additional attention. As health IT systems pass through different stages in their life cycle, it is key that the roles above are defined. Furthermore, the responsibilities associated with these roles and the importance of the information shared among these roles must also be understood.

During the transition from the Design and Development phase of the lifecycle to the Acquisition phase, the developer provides information to the system owner. This should be sufficient to allow the system owner to determine that the health IT software meets the needs of the Organization and can be used safely. The user and implementer may assist the system owner in making this determination but ultimately the onus is on the system owner to make the correct determination.

The system owner examines the planned use of IT in both the health IT system and the healthcare ecosystem during the transition from the Acquisition phase to the Installation and Integration phase. The need for configuration, customization, training of operators and users is also determined at this stage. Testing and monitoring of the system is also carried out.

During the transition from the Installation and Integration phase to the Implementation phase, the integrator provides information about any hazards that were identified during the integration. A mitigation strategy for hazards that are expected to be mitigated is put in place. The implementer provides any specific actions to the operator that are needed to maintain the safety of the health IT during its use in the health IT system during the transition from Implementation phase to the Operational use in the Clinical context phase. During the transition from Operational use in the Clinical Context phase to the Decommissioning phase, any hazards that may need special attention on decommissioning and disposal of the health IT software are identified.

Standards have been developed that address the stakeholder responsible for maintaining the key properties at various stages of the lifecycle. Due to the distribution of roles and responsibilities involved, the standards related to health IT infrastructure have provided a definition of the lifecycle and have developed standards for the stages of the lifecycle as follows:

• Design and Development Phase—addressed to Accountable Manufacturers
• Implementation Phase (which includes Acquisition, Installation and Implementation)—addressed mainly to Accountable Manufacturers and Accountable Healthcare Delivery Organizations
• Clinical Use Phase (which includes Operational Use, Maintenance and Decommissioning)—also addressed to Accountable Healthcare Delivery Organizations

In addition, foundational standards (such as ISO 81001-1) have been developed which provide terms, definitions, concepts, and core themes that are applicable across the life cycle and address specific aspects of the management of health IT systems such as Governance and Knowledge Transfer. Section 3.2 discusses standards addressed to Accountable Manufacturers and Section 3.3 discusses standards addressed to Accountable Healthcare Delivery Organizations and those related to Data Transfer. The interaction between standards and regulations is discussed in Section 3.4.

### 3.2. Standards for accountable manufacturers

In ensuring the safety, effectiveness and security of medical device and health software, there are a number of standards which accountable manufactures must implement. It should be noted that in the context of health IT systems, accountable manufacturers can be both medical device manufacturers who must comply with regulations in order to place their devices on the market, and to manufacturers of unregulated components of health IT systems such as electronic health record (EHR) systems which are not currently are not directly regulated (31). Standards relevant to both types of manufacturer are considered in this section.

Section 3 presents an overview of the healthcare ecosystem which includes: the *Health IT Infrastructure; the Broader Healthcare System and the Healthcare Delivery Context* used. In this section, from the *Health IT Infrastructure* perspective we examine the standards which are addressed to Healthcare Delivery Organizations and Accountable Manufacturers and examine how these standards are used to facilitate communication in order to ensure that the safety, effectiveness and security of the Health IT system are preserved. We also examine the standards that are used to ensure that data can be transferred between Health IT systems. In the context of *the Broader Healthcare System*, we examine the relationship between regulations and the harmonized standards and also examine other regulations and directives that have a bearing on the effectiveness of digital implementation of connected health solutions. A summary of the standards and regulations is provided in Table 1.

**Table 1:** Summary of healthcare standards and regulations.

Name/Number:	Standard—health IT infrastructure	Regulation—broader healthcare system	Directive—broader healthcare System	Design and development:	Implementation:	Clinical use:	Data transfer	Accountable medical device manufacturer	Accountable health software manufacturer	Healthcare delivery organization
ISO 13485:2016	✓			✓				✓		
ISO 14971:2019	✓			✓				✓		
IEC 80001-1:2021	✓			✓	✓	✓		✓	✓	✓
ISO 81001-1:2021	✓			✓	✓	✓		✓	✓	✓
IEC 60601-1: 2012	✓			✓				✓		
IEC 62304:2006	✓			✓				✓		
ISO 13606-1:2019	✓					✓	✓			
FHIR	✓					✓	✓			
DICOM	✓					✓	✓			
Medical devices regulation (MDR)		✓		✓				✓		
*In vitro* diagnostic devices (IVDR)		✓		✓				✓		
General data protection regulation (GDPR)		✓		✓	✓	✓	✓	✓	✓	✓
EU accessibility directive			✓	✓	✓	✓	✓	✓	✓	✓

#### 3.2.1. Standards for medical device manufacturers

##### 3.2.1.1. Quality management system and risk management standards

*ISO 13485:2016 Medical devices—Quality management systems—Requirements for regulatory purposes* (32) is a quality management system standard which “specifies requirements for a quality management system where an organization needs to demonstrate its ability to provide medical devices and related services that consistently meet customer and applicable regulatory requirements”. The standard states that organizations can be involved in one or more stages of the life-cycle, including design and development, production, storage and distribution, installation, or servicing of a medical device and design and development or provision of associated activities (e.g., technical support) and as such the information from this management system standard will have relevance throughout the lifecycle of the health IT infrastructure as defined in ISO 81001-1:2021 though published prior to it.

*ISO 14971:2019 Medical devices—Application of risk management to medical devices* (33) is a risk management standard which specifies terminology, principles and a process for risk management of medical devices, including software as a medical device and *in vitro* diagnostic medical devices. The process described in this document intends to assist manufacturers of medical devices to identify the hazards associated with the medical device. The standard also assists manufacturers in estimating and evaluating the associated risks, to control these risks, and to monitor the effectiveness of the controls. Under the standard, accountable medical device manufacturers must take a life cycle approach to risk management. They must document and maintain a risk management process to identify hazards associated with a medical device, estimate and evaluate the associated risks, implement risk control measures for these risks and monitor the effectiveness of the controls which have been implemented. This process must include risk analysis, risk evaluation, risk control and evaluation of residual risk.

*IEC 80001-1:2021 Application of risk management for IT-networks incorporating medical devices—Part 1: Safety, effectiveness and security in the implementation and use of connected medical devices or connected health software* (34) outlines the roles, responsibilities and activities that are required to complete this risk management process of the health information technology system of which the medical device is a component. This standard is discussed in detail in Section 3.3 as it is addressed to accountable healthcare delivery organizations. IEC 80001-1 is aligned with ISO 14971. All documentation produced by the medical device manufacturer as a result of the performance of risk management activities during the development of the medical device must be maintained in the risk management file. The medical device manufacturer must also document the intended use and reasonably foreseeable misuse of the medical device as well as known and foreseeable hazards associated with the medical device in both normal and fault conditions. This information must also be maintained in the risk management file and will feed into the risk management process undertaken by healthcare delivery organizations under the requirements of IEC 80001-1.

##### 3.2.1.2. Medical device software standards

ISO 13485 is often seen as the first step in obtaining certification and CE mark for medical devices (35). CE marking is the medical device manufacturer's claim that a product meets the General Safety and Performance Requirements (GSPR) of all relevant European Medical Device Regulations. Medical devices must have a CE mark to be placed on the market in the European Union (36, 37). However, the quality management system requirements outlined in ISO 13485 are not strictly related to software development issues. To address these issues, *IEC 62304:2006 Medical device software—Software life-cycle processes* (38) defines the life cycle requirements for medical device software. The standard outlines the set of processes, activities, and tasks and establishes a common framework for medical device software life cycle processes. IEC 62304 is a harmonized standard. Harmonized standards, such as IEC 62304 and ISO 13485, and their relationship to regulations are discussed in Section 3.5.

##### 3.2.1.3. Medical electrical equipment standards

To ensure the safety, effectiveness and security of the health information technology system, there is a requirement to have a robust risk management process in place throughout the lifecycle. The roles, responsibilities and activities that are required to complete this risk management process are outlined in IEC 80001-1 which, while addressed mainly to accountable Healthcare Delivery Organizations, also requires other stakeholders, who are external to the healthcare delivery organization, to participate in the risk management process. External participants in the risk management process include accountable medical device manufacturers and accountable manufacturers of other health software. IEC 80001-1 requires that accountable medical device manufacturers provide Healthcare Delivery Organizations with accompanying documents describing the intended use of a medical device and providing instructions for the safe installation and use of the device. Accountable manufacturers of other health software must make documents available to the Healthcare Delivery Organization. This documentation should provide information for the safe use of the technology provided on the IT network including, but not restricted to: technical descriptions and manuals; required IT network characteristics; and known incompatibilities and restrictions. The information required from medical device manufacturers is currently provided based on their responsibilities outlined under *IEC 60601-1* (39). This standard contains requirements for basic safety and essential performance applicable to medical electrical equipment. IEC 80001-1 does not impose any additional requirements on manufacturers in term of the provision of information. However, the standard does require that this information be aggregated and used within the risk management process (40, 41).

#### 3.2.2. Standards for manufacturers of health software

Health IT system infrastructure includes both regulated medical devices and other health software that is not regulated. Standards such as IEC 80001-1 place certain responsibilities on Accountable providers of Health Software (referred to in IEC 80001-1 as a “Health Software Manufacturer” and recognised as an external risk management stakeholder along with medical device manufacturers). The standard requires that Health Software Manufacturers and medical device manufacturers provide accompanying documents that can be used by the Healthcare Delivery Organization to identify hazards “associated with deployment of the health IT system and its use under both normal and foreseeable operating conditions”. In addition to these standards, medical device manufacturers, manufacturers of health software, and Healthcare Delivery Organizations must consider the standards that are associated with the communication of health data. These standards are discussed in Section 3.3.

### 3.3. Standards for healthcare delivery organizations

For Healthcare Delivery Organizations, there are also a number of standards that have been developed in order to ensure the safety, effectiveness and security of the health IT system during the Implementation and Clinical Use Phase. The main focus of these standards is on providing healthcare delivery Organizations with requirements for the roles, responsibilities and activities that need to be in place in order to manage the risk associated with “the implementation and use of connected medical devices or connected health software” (34). The previous version of *IEC 80001-1* published in 2010 (30) was developed in response to reports of a number of cyber-attacks on US hospitals after which guidance on the prevention of such attacks was published (42). This led to a review of the risks surrounding networked medical devices and the standard was developed to address these risks.

The publication of IEC 80001-1 recognised that, while these regulations address the operation of the device prior to acquisition by the Healthcare Delivery Organization, the placement of the device into a network creates a shared responsibility for the risk management of the device between the accountable manufacturers and the Healthcare Delivery Organization (43, 44). While medical device manufacturers are used to implementing this type of standard, Healthcare Delivery Organizations have struggled to implement the requirement of the standard and research has been performed to address these challenges (45–47). This research included the development of a technical report to allow for the assessment of the capability of risk management processes (48) and the proposal of an approach to the revision of the standard to make it easier to understand and implement (49).

The new version of the standard recognizes that Health IT infrastructures have evolved since the initial standard was developed. Where the original standard established a concept of a medical IT network as a network incorporating at least one medical device, the new standard looks at a broader scope of connected medical devices and connected health software. Therefore, it recognizes that Health IT infrastructures are increasingly connected, medical devices are routinely placed onto the hospital's general IT network and that the Health IT infrastructure increasingly reaches beyond traditional care settings. This increases the potential for adverse events as medical devices may be placed into systems where they have not previously been tested. Therefore, there must be a greater emphasis on the risk management process for these devices and greater emphasis on Healthcare Delivery Organizations to understand and manage these risks.

In addition to the 2010 version of the IEC 80001-1 standard, a number of associated *technical reports* were published. The technical reports provide guidance to address specific aspects of the implementation of the IEC 80001-1 standard. The standard, along with the associated technical reports, can be used to manage the communication between the healthcare delivery organizations throughout the lifecycle to ensure that the safety, effectiveness and security of connected medical devices and connected health software are preserved. A number of these technical reports are currently being reviewed and revised. In addition, a foundational standard, *ISO 81001-1* has been published to define “the principles, concepts, terms and definitions for health software and health IT systems, key properties of safety, effectiveness and security, across the full life cycle, from concept to decommissioning” (22).

### 3.4. Standards for data transfer

The establishment of the types of interconnected health IT infrastructures has established a need to transfer data across and between these systems and often across geographical regions. In order to ensure the integrity of the data being transferred, a number of standards have been established. This transfer of data requires robust communication among Healthcare Delivery Organizations and accountable manufacturers and the risks associated with the transfer of this data would be managed within the broader context of risk management under the requirements of IEC 80001-1 and would also need to be managed in the context of the regulations pertaining to data protection. These regulations are discussed in the context of the broader healthcare system in Section 3.5. Some standards that would be relevant in this context include *ISO 13606-1:2019 Health informatics—Electronic health record communication—Part 1: Reference model* (50). This standard specifies a means for communicating part or all of the electronic health record (EHR) of one or more identified subjects of care between EHR systems, or between EHR systems and a centralized EHR data repository. Fast Interoperability Resources (FHIR) is a Health Level Seven International (HL7) specification for healthcare interoperability (51) and “is designed to enable health data, including clinical and administrative data, to be quickly and efficiently exchanged” (52). For the transfer of medical images, the Digital Imaging and Communications in Medicine (DICOM) standard is used. DICOM (53) is “the international standard to transmit, store, retrieve, print, process and display medical imaging information”. These standards need to be considered in the context of the other standards—both those addressed to Healthcare Delivery Organizations and accountable manufacturers and in the context of the overall healthcare ecosystem.

### 3.5. The broader healthcare system—regulations

The broader healthcare system encompasses for example, regulations, funding and policy implications, within which the Health Delivery Organization must comply and operate. This section examines the broader healthcare system from the perspective of the regulations that impact the system at various stages of the Health It system lifecycle. The regulations examined in this paper are focused on those which are applicable in the EU.

As previously stated health software is not currently regulated (unless it is classified as a medical device) and, as such, during the design and development phase, we focus on the regulations that are relevant to medical devices. Medical Devices that are designed to be marketed in the EU must comply with *Regulation 2017/745* on Medical Devices (MDR) (54) and *Regulation 2017/746* on *In Vitro* Diagnostic Devices (IVDR) (55). These regulations became applicable after a 5-year transition period and represent a significant development and strengthening of the existing regulatory system for medical devices in Europe. The legislation now is in the form of a Regulation, rather than a Directive, which means that the EU law is directly applicable at national level meaning that there is no longer a requirement for transposition through specific national legislation which should prevent variation in the approach taken.

The EU also states that for the new regulation that “Compliance with a harmonized standard confers a presumption of conformity with the corresponding essential requirements set out in Union harmonization legislation from the date of publication of the reference of such standard in the Official Journal of the European Union” (56). This means that manufacturers that comply with the requirements of the recognised standards can also claim conformity to the regulations. To date, 14 standards have been recognised and it is expected that the Commission will issue further implementing decisions to add to the list of harmonized standards later in 2022. Some standards (such as IEC 62304:2006 Medical device software—Software life cycle processes) which conferred a presumption of conformity with the previous Medical Device Directive have not yet been recognised. This standard was discussed in Section 3.2.1.

During the Implementation Phase, Health Delivery Organizations will also need to consider regulation related to the data that is being transmitted. Privacy issues will also need to be addressed. In the EU, the General Data Protection Regulation (GDPR) (55) recognizes data concerning health as a special category of data and provides a definition for health data for data protection purposes. It requires specific safeguards for personal health data which will need to be addressed in the context of connected health, including the facilitation of cross border care.

In May, 2022, the European Commission published a proposal for a Regulation on the European Health Data Space (EHDS) (57). With the proposal, the European Commission aims to make significant progress towards a single market for digital health services and products with the overall objective being to ensure that electronic health data are as open as possible and as closed as necessary to facilitate research, innovation, policy-making, and regulatory activities. The aim is to have a single internal market for health data between the EU Member States.

The Clinical Use phase consists of Operational Use, Maintenance and Decommissioning. The focus for both the medical device manufacturer and the Health Delivery Organization is to ensure that the connected health system continues to be compliant with the relevant regulations and standards as these activities take place. For example, when making a change to a device within an existing system, in order to address a security vulnerability, the manufacturer and Health Delivery Organization will need to ensure that the change is made within the existing risk management process and that the change does not impact the key properties of the system.

Connected Health systems are increasingly including Medical Devices that use sophisticated Artificial Intelligence. The European Commission published its Proposal for a Regulation on Artificial Intelligence (AI) (58) in April of 2021, which aims to develop a comprehensive framework for the regulation of AI. Parts of the proposal address high risk AI applications, which would include the use of AI in Medical Devices and Connected Health systems. No international guidance, common specifications and/or harmonized standards currently exist for the use of AI in medical devices. Therefore, regulators continue to work to address the challenge of regulation of medical device software that include AI algorithms and to address the unique challenges that AI can give rise to in the context of healthcare including, for example, the issues related to the automated processing of data and compliance with GDPR which requires that “meaningful information about the logic” involved in decisions related to their care is provided by manufacturers to patients.

Given that we are concerned about the effectiveness of Connected Health system, another directive that we should be concerned with when developing Health IT systems is the EU Accessibility directive, EN 301 549 V3.2 (59). This came into effect in June 2021. This directive requires that all public sector bodies in the EU have accessible online websites and mobile apps, through which many connected health solutions are implemented. In their research, Tsvyatkova et al. (60), present accessibility as having concern for the quality of being “easy to reach and use”.

We are aware that the directive, EN 301 549, has not been published for medical devices specifically, which is one of the reasons that we want to highlight it here. Many health IT systems, and specifically those used in a connected health context, will be developed for use by the public (patient, family, carers, healthcare professionals), and, consequently, should be accessible. Health IT system users will often have accessibility issues through disability, impairment or limitation, for example, visual impairment, intellectual and developmental disability. Designers and developers should ensure that the software they develop provides correct functions for the user. Good design will also ensure accessible interaction through a user interface which would include, for example, features which support new users in understanding and using the software. Furthermore, designing of interactive elements which support low physical effort should be considered.

Additionally, while the directive is written for public bodies, and which includes many national health services in European countries, should accessibility not be considered by all, regardless of whether they are public or private? The directive is aligned to the Web Content Accessibility Guidelines v2.1, published by the W3C and known as WCAG 2.1 (61). These are internationally recognised requirements for producing web and mobile content, are considered best practice, and are very widely used. It should be noted that the directive also contains requirements not mentioned in WCAG 2.1, and so, there should not be a singular reliance on WCAG 2.1 when developing accessible software.

## 4. The healthcare delivery context– clinical change management

Implementing technology into the healthcare ecosystem requires consideration of change management not only in the context of the technology itself, but also from the perspective of each Healthcare Delivery context and for each the other Connected Health elements—defined healthcare pathways, healthcare professionals, patients and/or carers, standards and regulations, and health data sharing. While we have focused on Standards and Regulations in this paper, we now consider the need for clinical change management to support and the integration of connected health solutions in these Organizational settings.

### 4.1. Clinical change management

ISO 81001 defines clinical change management as:

“a strategic and systematic approach that supports people and their organizations in the successful transition and adoption of electronic health solutions, with a focus on outcomes including solution adoption by users and the realization of benefits” (22)

This definition is sourced from a guide “A Framework and Toolkit for Managing eHealth Change: People and Processes” that has been developed by Canada Health Infoway (62), an independent, not-for-profit organization funded by the Canadian federal government. This framework identifies six core elements of the Clinical Change Management process as follows:

1. Governance & Leadership
2. takeholder Engagement
3. Communications
4. Workflow Analysis & Integration
5. Training & Education
6. Monitoring & Evaluation

ISO 81001 also notes that Health IT systems implementation usually necessitates changes in clinical and business workflows and advocates that these changes should be managed through a comprehensive clinical change management process which contains these six elements. The standard notes that a comprehensive clinical change management process “will ensure appropriate clinical and business input in designing, monitoring and optimizing the process (including a suitable post-implementation period to maximize effectiveness and minimize safety and security risk)”. In this section, we examine how each of the six elements of the clinical change management process must be addressed in a given Healthcare Delivery Context. Our focus is on understanding how each element contributes to ensuring the safety, effectiveness and security of the Health IT system including the impact of the element on the successful implementation of the standards (and regulations) discussed in Section 3. The remainder of this section discusses each of these elements in this context.

#### 4.1.1. Governance and leadership

##### 4.1.1.1. Change management

In addressing why this element was selected, the Infoway Guide (62) states that “Without an effective governance structure, the strategic view that links project tasks together—the ‘what are we doing’ and ‘why are we doing it,’ never gets answered and the project risks loss of aim, direction and successful execution”. It is also noted that without this support for change that there is a risk that damage to the organizational culture, leads to a lack of buy in from stakeholders which ultimately results in the failure of the project. Without leadership support and a robust change management process in place, change will happen in an ad hoc manner, but will not be sustainable. It is needed to ensure that the other elements described below will happen. Governance and Leadership are defined within the Infoway Guide (63) as:

“The mechanisms used to guide, steer or regulate the course of a project, including how stakeholders can affect the priorities and progress of a project as well as the Change Management activities occurring within a project.”

Governance and leadership within the Clinical Change Management Process will ensure that, for the specifics of their Healthcare Delivery Context, the process runs correctly, making it sustainable through workable process definitions. They must also analyze how well the process is working, and implement change when it is needed.

##### 4.1.1.2. Standards

In addressing the key properties of safety, effectiveness and security, the IEC 80001-1 refers to the Leadership team within a Healthcare Delivery Organization as “Top Management” and notes “that effective risk management depends on its integration with the governance of the organization, including decision-making” and that it “is the responsibility of the Top Management of the Organization to ensure that risk management is implemented throughout the Health IT System lifecycle, and that its effectiveness is evaluated”.

#### 4.1.2. Stakeholder engagement

##### 4.1.2.1. Patient centered care

The current view of the Healthcare system increasingly places the patient at the center with care being provided by a care team which includes family members who act as caregivers, healthcare professionals and others. This care is provided within a Healthcare Delivery Organization (which increasingly includes care in the community) and the Organization operates within a larger environment which includes the regulatory, market and policy framework (64). Increasingly, it is recognized that the correct stakeholders need to be included in any change. For effective Connected Health implementation, different diverse stakeholder groups from within the Healthcare System need to be consulted at different stages, providing their input into each of the six elements of clinical change management.

##### 4.1.2.2. Standards and regulations

From a standards perspective, stakeholder engagement is vital to supporting safe, effective and secure Connected Health systems. Stakeholder engagement is required to support the risk management process when accountable manufactures provide information to allow Health Delivery Organizations to integrate these components into the Health IT infrastructure (2). This engagement is needed through the lifecycle and is also required from a regulatory perspective through the implementation and outputs from the implementation of harmonized standards. The collection and transmission of data requires stakeholder engagement in understanding what data is required from a clinical perspective and, in the case where the transfer of this data is required, to ensure that consent has been obtained from the patient for this transfer to take place.

In addition, for the effective use of Connected Health systems, we have discussed how the Accessibility directive is important. While the directive provides useful and relevant guidance to the designer and developer, bringing a variety of stakeholders on board will support the practical development of systems which should be accessible for all.

#### 4.1.3. Communications

##### 4.1.3.1. Standards

As Health IT system can be composed of diverse technologies, both regulated and unregulated, Healthcare Delivery Organizations require specific information from Accountable manufacturers in order to allow the combination of these technologies in a way that continues to be safe, effective and secure. ISO 81001-1 notes that “A culture of safety involves continuous communication, education and awareness building on evidence involving continuous monitoring, documentation and analysis”. The ongoing process of clinical change management must facilitate this culture and ensure that this level of ongoing communication persists throughout the complex lifecycle of the health IT infrastructure. Ongoing communication is also required to support the changes to business and clinical workflow that are necessitated by the implementation of Health IT system. While the standards mainly focus on the relationship between the Healthcare Delivery Organization and the Accountable Manufacturers, it is also noted that a diverse team of risk management stakeholders from within the Healthcare Delivery Organization to support the risk management and clinical change management processes (48). In order to gain a holistic view of the risk management process for the specific context in which the Healthcare Delivery Organization operates, care must be taken to facilitate communication among these stakeholders (47). In addition, it should be noted that stakeholders have different perspectives based on their roles and the process should aid communication to better understand these differing perspectives (65).

#### 4.1.4. Workflow analysis and integration

##### 4.1.4.1. Changing care pathways

Care pathways are set up in healthcare to standardize the method of care (66) and have become a main tool used to manage healthcare quality (67), expanding from primary and secondary care into the community. And, we have seen particularly during the Covid-19 pandemic, how care homes, hospices and pharmacies are all implementing Connected Health systems. However, due to the implementation of Connected health, new clinical pathways and care delivery mechanisms need to be defined, taking Health IT into account. What is important is that the people implementing these Health IT systems to follow modified care pathways are aware of the relevant standards and regulation, and that they ensure that the infrastructure implemented is following these standards and regulations.

#### 4.1.5. Training and education

##### 4.1.5.1. Standards

The Infoway Guide (63) refers to training as “The act of imparting both knowledge and specific skills among key stakeholders to promote adoption.” IEC 80001-1 notes that as part of the completion of risk management activities, prior to the deployment of the Health IT system for live use that it is possible that existing workflows will need to be adjusted to accommodate the Health IT System and that Operators and Users will need to be trained. ISO 81001-1 further notes that “Assigned resources require the education, training and time to apply the level of effort and skill necessary to carry out safety, security and effectiveness activities in a robust and competent manner”.

#### 4.1.6. Monitoring and evaluation

##### 4.1.6.1. Standards

The Infoway Guide defines monitoring and evaluation as “the process of reviewing whether Change Management activities took place as planned; and the extent to which they were effective.” In the context of IEC 80001-1, the risk management framework includes “Evaluation” and “Improvement” During the Evaluation Phase, the standard requires that “compliance to the Risk Management Plan and effectiveness of the risk management Process should be periodically evaluated” with a view to improving the “suitability, adequacy and effectiveness of the risk management process and the way that the risk management process is implemented”.

### 4.2. Future connected health systems

This paper has examined the complexity of current Health Information Technology systems and the approaches that are currently taken to ensuring their safety, effectiveness and security. These systems are evolving and Artificial Intelligence is increasingly becoming part of the Health IT infrastructure. Regulations and standards for the use of AI in Healthcare Systems is currently being developed and in particular machine-learning-based models present a unique challenge to regulatory agencies. These models can evolve rapidly as more data and user feedback are collected and it is not currently clear how regulators can evaluate these changes (68). In addition, artificial intelligence can be used to infer health status from the collection of data from wearable devices. These devices may not be regulated which can compromise the accuracy of the data that is being collected (69) which impacts the accuracy of the results from these systems. Standards development efforts in this area can be seen in the remit and current work of ISO/IEC JTC 1/SC 42 (70). Current standards and regulations need to evolve to address the potential impact of the use of AI in healthcare systems and the implementation of these systems will need to be managed as part of an updated Clinical Change Management process. Updated clinical change management processes need to be cognizant of the changes and increased focus on risk management that the implementation of these systems necessitate.

## 5. Conclusion

Our focus in this paper has been on Connected Health, and, in particular, the importance of Standards and Regulations in supporting the implementation of safe, effective and secure Connected Health solutions. Standards define the healthcare ecosystem as being composed of: the *Health IT Infrastructure; the Healthcare Delivery Context and the Broader Healthcare System*. The implementation of Connected Health solutions within this ecosystem requires consideration of all of these components as none can exist in isolation. However, if any one of these is not considered, then there is a very high risk that the Connected Health solution will not work in practice.

For these purposes, the Health IT infrastructure must be developed taking standards and regulations, which form part of the *Broader Healthcare System* into account. At one level, this needs to be done for ratification by the relevant bodies globally—Notified Bodies in the European Union or by the Food and Drugs Administration in the US. More importantly, these regulations are one means of supporting development which is safe, effective and secure for the Connected Health solution user. The standards and regulations exist out of concern for the welfare of the user, and must take them into account. For example, in cases where the accessibility directive is not implemented, then those with a disability are immediately not catered for. Data is an important component and those whose data is stored in Connected Health Systems want to be satisfied that their data is secure. In order to implement the requirements of the standards related to the implementation and clinical use of the Health IT infrastructure, close collaboration and ongoing communication between Healthcare Delivery Organizations and Accountable Manufacturers is required throughout the lifecycle. Within Healthcare Delivery Organizations, teams of diverse risk management stakeholder groups must be convened. They should be assisted in communication supporting the development of a holistic view of the risk management process in the specific *Healthcare Delivery Context* in which the Health IT infrastructure operates.

We have shown how standards and regulations can support this, but it should be noted that there are often other non-specified technical solutions which can be used. Implementation of Connected Health solutions, which requires bringing technology into the healthcare system requires a robust and comprehensive approach to *Clinical Change Management* to support the business and clinical changes that the implementation of such solutions requires. Change managers can look to ISO 81001-1, which identifies six elements of the Clinical Change Management Process as follows: Governance and Leadership, Stakeholder Engagement, Communications, Workflow Analysis and Integration, Training and Education, and Monitoring and Evaluation. Each of these elements are vital to the implementation of standards and regulations and in facilitating the non-technical changes required. For example, as technology is introduced into healthcare system, healthcare pathways must change. These can be re-defined by healthcare professionals, but if they do not consider standards and regulations, they are in danger of losing some of the positive effects of making these changes.

Ultimately, to implement safe, effective and secure Connected Health solutions in the healthcare ecosystem, it requires that all those involved work together to bring the identified components together, standards and regulations, health IT, people, data, and healthcare pathways, thus resulting in the central requirement—patient-centered care.

## Footnotes

^1^A “medical device” is defined in the EU Medical Device Regulations (MDR) as “any instrument, apparatus, appliance, software, implant, reagent, material or other article intended by the manufacturer to be used, alone or in combination, for human beings for one or more of the following specific medical purposes:

• diagnosis, prevention, monitoring, prediction, prognosis, treatment or alleviation of disease,
• diagnosis, monitoring, treatment, alleviation of, or compensation for, an injury or disability,
• investigation, replacement or modification of the anatomy or of a physiological or pathological process or state,
• providing information by means of *in vitro* examination of specimens derived from the human body, including organ, blood and tissue donations, and which does not achieve its principal intended action by pharmacological, immunological or metabolic means, in or on the human body, but which may be assisted in its function by such means.”

^2^Medical Device interoperability has been defined by the FDA as “the ability to safely, securely, and effectively exchange and use information among one or more devices, products, technologies, or systems. This exchanged information can be used in a variety of ways including display, store, interpret, analyze, and automatically act on or control another product.”

## References

[1] Kihlstrom G. The importance of aligning people, processes and technology amid transformation initiatives, Forbes (2022). Available at: https://www.forbes.com/sites/forbesagencycouncil/2022/03/21/the-importance-of-aligning-people-processes-and-technology-amid-transformation-initiatives/?sh=3b2856a26ba6 (Accessed August 25, 2023).

[2] MacMahon ST Richardson I. Regulating connected health: pathways, technology and the Patient, TechREG Competition Policy International (2022).

[3] Wagner EH Austin BT Davis C Hindmarsh M Schaefer J Bonomi A. Improving chronic illness care: translating evidence into action. Health Aff. (2001) 20(6):64–78. 10.1377/hlthaff.20.6.64 11816692

[4] Rangachari P Mushiana SS Herbert K. A narrative review of factors historically influencing telehealth use across six medical specialties in the United States. Int J Environ Res Public Health. (2021) 18(9):4995. 10.3390/ijerph18094995 34066829 PMC8125887

[5] Hoffman C Rice D. Chronic care in America: A 21st century challenge. Princeton, NJ: The Robert Wood Johnson Foundation (1996).

[6] Wagner EH. The role of patient care teams in chronic disease management. Br Med J. (2000) 320(7234):569. 10.1136/bmj.320.7234.569 10688568 PMC1117605

[7] Institute of Medicine. Crossing the quality chasm: a new health system for the 21st century, a new health system for the 21st century. Edited by Rona Briere, National Academy of Sciences (2001).

[8] Lee I Pappas GJ Cleaveland R Hatcliff J Krogh BH. High-confidence medical device software and systems. Computer. (2006) 39(4):33–8. 10.1109/MC.2006.180

[9] Hamilton A Nau R Burke R Weinstein S Dlatt CKB Fiore S Conyers JL. Summary of the August 2011 symposium on the role and future of health information technology in an era of health care transformation. The George Washington University (2011).

[10] West Health Institute. The value of medical device interoperability - improving patient care with more than $30 billion in annual health care savings (2013).

[11] Centers for Medicare & Medicaid Services. Medicare and medicaid programs; electronic health record incentive program; final rule. Edited by Health and Human Services (2010).

[12] Centers for Medicare & Medicaid Services. EHR incentive programs (2013). Available at: http://www.cms.gov/Regulations-and-Guidance/Legislation/EHRIncentivePrograms/index.html?redirect=/ehrincentiveprograms

[13] Milenkovich N. OCR issues new HITECH regulations. Drug topics - voice of the pharmacist (2013). Available at: http://drugtopics.modernmedicine.com/drug-topics/news/drug-topics/health-system-news/ocr-issues-new-hitech-regulations

[14] Castañeda M. Connecting devices and data on the healthcare network. Biomed Instrum Technol. (2010) 44(1):18–25. 10.2345/0899-8205-44.1.18 20374114

[15] Agency for Healthcare Research and Quality (AHRQ). Health IT for improved chronic disease management. Edited by Department of Health and Human Services (2013).

[16] Comstock J. http://mobihealthnews.com/28295/14m-networked-medical-devices-to-ship-by-2018/.

[17] Peek N Sujan M Scott P. Digital health and care in pandemic times: impact of COVID-19. BMJ Health Care Inform. (2020) 27(1):2–4. 10.1136/bmjhci-2020-100166 PMC7307522 32565418

[18] Porter S. The impact of COVID-19 on health tech adoption in the UK, Healthcare IT News (2020). Available at: https://www.healthcareitnews.com/news/emea/impact-covid-19-health-tech-adoption-uk (Accessed September 14, 2022).

[19] Furtner D Shinde SP Singh M Wong CH Setia S. Digital transformation in medical affairs sparked by the pandemic: insights and learnings from COVID-19 era and beyond. Pharmaceut Med. (2022) 36(1):1–10. 10.1007/s40290-021-00412-w 34970723 PMC8718376

[20] Lee CH Wang D Desouza KC Evans R. Digital transformation and the new normal in China: how can enterprises use digital technologies to respond to COVID-19? Sustainability. (2021) 13(18):10195. 10.3390/su131810195

[21] Baudier P Kondrateva G Ammi C Chang V Schiavone F. Digital transformation of healthcare during the COVID-19 pandemic: patients’ teleconsultation acceptance and trusting beliefs. Technovation. (2023) 120:102547. 10.1016/j.technovation.2022.102547

[22] ISO. ISO 81001-1: health software and health IT systems safety, effectiveness and security—part 1: principles and concepts. Geneva, Switzerland (2021).

[23] Jen MY Kerndt CC Korvek SJ. Health information technology. St. Petersburg, FL: StatPearls Publishing (2022). 29262233

[24] Whitehead SF Goldman JM. Getting connected for patient safety how medical device “plug-and-play” interoperability can make a difference, patient safety and quality healthcare (2008).

[25] Venkatasubramanian KK Gupta S Jetley RP Jones PL. Interoperable medical devices - communication security issues. IEEE Pulse. (2010).

[26] Graham J Dizikes C. Baby’s death spotlights safety risks linked to computerized systems. Chicago: Chicago Tribune (2011).

[27] National Cybersecurity and Communications Integration Center. Attack surface: healthcare and public health sector (2012).

[28] Talbot D. Computer viruses are “Rampant” on medical devices in hospitals, MIT Technology Review (2012).

[29] ISO/IEC. ISO/IEC guide 2:2004 standardization and related activities—general vocabulary (2004). Available at: https://www.iso.org/standard/72026.html (Accessed September 16, 2022).

[30] IEC. IEC 80001-1 - application of risk management for IT-networks incorporating medical devices - part 1: roles, responsibilities and activities. Geneva, Switzerland: International Electrotechnical Commission (2010).

[31] Konnoth C. Are electronic health records medical devices? Cambridge, UK: Cambridge University Press (2016). 36–46.

[32] ISO. ISO 13485:2016 medical devices – quality management systems – requirements for regulatory purposes. Geneva, Switzerland (2016).

[33] ISO. ISO 14971:2019 - medical devices - application of risk to medical devices. Geneva, Switzerland: International Organisation for Standardization (2019).

[34] IEC. IEC 80001-1:2021 application of risk management for IT-networks incorporating medical devices—part 1: safety, effectiveness and security in the implementation and use of connected medical devices or connected health software (2021).

[35] Bujok AB MacMahon ST McCaffery F Whelan D Mulcahy B Rickard WJ. Safety critical software development – extending quality management system practices to achieve compliance with regulatory requirements. In: Clarke P O'Connor R Rout T Dorling A, editors. Communications in computer and information science. Vol 609. Cham: Springer (2016); Software Process Improvement and Capability Determination. SPICE 2016.

[36] National Standards Authority of Ireland (NSAI). CE marking for medical devices. NSAI (2009). Available at: http://www.nsai.ie/Our-Services/Certification/Medical-Devices/CE-Marking-for-Medical-Devices.aspx

[37] British Standards Institution. CE Marking, bsi (2023). Available at: https://www.bsigroup.com/en-GB/medical-devices/our-services/ce-marking/ (Accessed August 25, 2023).

[38] IEC. IEC 62304:2006 medical device software – software life cycle processes. Geneva, Switzerland (2006).

[39] IEC. IEC 60601-1 - medical electrical equipment - part 1: general requirements for basic safety and essential performance (2012).

[40] Cooper T Eagles S. 80001: new era dawns for medical devices. Biomed Instrum Technol. (2011) 45(1):16–25. 10.2345/0899-8205-45.1.16 21322804

[41] Eagles S. Medical device software standards for safety and regulatory compliance (2013).

[42] U.S. Department of Health and Human Services, et al. Guidance for industry - cybersecurity for networked medical devices containing off-the-shelf (OTS) software. Edited by Food and Drug Administration. 5630 Fishers Lane, Room 1061, (HFA-305), Rockville, MD, 20852 (2005).

[43] Cooper T David Y Eagles S. Getting started with IEC 80001: Essential information for healthcare providers managing medical IT-networks. Arlington, VA: AAMI (2011).

[44] Fuchs K. Using risk management to successfully deploy wireless medical devices. Medical Product Manufacturing News, 29(1) (2013).

[45] MacMahon ST McCaffery F Eagles S Keenan F Lepmets M Renault A. Development of a process assessment model for assessing medical IT networks against IEC 80001-1. In: Mas A Mesquida A Rout T O’Connor RV Dorling A, editors. Communications in computer and information science. Vol 209. Berlin, Heidelberg: Springer (2012); Software Process Improvement and Capability Determination. SPICE 2012. p. 148–60.

[46] MacMahon ST McCaffery F Lepmets M Eagles S. Assessing against IEC 80001-1. Healthinf 2013; Barcelona, Spain (2013). p. 305–308

[47] Hegarty FJ MacMahon ST Byrne P McCaffery F. Assessing a hospital’s medical IT network risk management practice with 80001-1. Biomed Instrum Technol. (2014) 48(1):64–71. 10.2345/0899-8205-48.1.64 24548041

[48] ISO. ISO/TR 80001-2-7: 2015 - application of risk management for IT-networks incorporating medical devices – application guidance – part 2-7: guidance for healthcare delivery organizations (HDOs) on how to self-assess their conformance with IEC 80001-1 (2015). p. 67–72.

[49] MacMahon ST Cooper T McCaffery F. Revising IEC 80001-1: risk management of health information technology systems. Comput Stand Interfaces. (2018). 10.1016/j.csi.2018.04.01333311855

[50] ISO. ISO 13606-1:2019 health informatics—electronic health record communication—part 1: reference model (2019).

[51] HL7. HL7 FHIR release 4B (2022). Available at: https://hl7.org/FHIR/

[52] The Office of the National Coordinator for Health Technology. What is HL7 ® FHIR ®?, 7, pp. 1–2 (2012).

[53] NEMA. Digital imaging and communications in medicine (2022). Available at: https://www.dicomstandard.org/

[54] The European Parliament and the Council of the European Union. Regulation (EU) 2017/745 of the European parliament and of the council of 5 April 2017 on medical devices, amending DIRECTIVE 2001/83/EC, REGULATION (EC) No 178/2002 and regulation (EC) No 1223/2009 and repealing council directives 90/385/EEC and 93/42/EE (2017).

[55] European Council. Regulation (EU) 2016/679 of the European Parliament and of the Council of 27 April 2016 on the protection of natural persons with regard to the processing of personal data and on the free movement of such data, and repealing directive 95/46/EC, EUR-Lex (2016). Available at: https://eur-lex.europa.eu/eli/reg/2016/679/oj (Accessed July 27, 2022).

[56] European Council. Commission implementing decision (EU) 2021/1182 of 16 July 2021 on the harmonised standards for medical devices drafted in support of regulation (EU) 2017/745 of the European Parliament and of the Council, EUR-Lex (2021). Available at: https://eur-lex.europa.eu/eli/dec_impl/2021/1182/oj (Accessed July 27, 2022).

[57] European Council. European health data space, European commision (2021). Available at: https://health.ec.europa.eu/ehealth-digital-health-and-care/european-health-data-space_en (Accessed July 27, 2022).

[58] European Council. Proposal for a regulation of the European parliament and of the council laying down harmonised rules on artificial intelligence (artificial intelligence act) and amending certain union legislative ACTS, EUR-Lex (2021). Available at: https://eur-lex.europa.eu/legal-content/EN/TXT/?uri=CELEX%3A52021PC0206 (Accessed July 27, 2022).

[59] European Union. Directive (EU) 2016/2102 of the European parliament and the council of 26 October 2016 on the accessibility of the websites and mobile applications of public sector bodies, EN 301 549 V3.2.1, web accessibility directive (2016). Available at: https://eur-lex.europa.eu/legal-content/EN/TXT/?uri=CELEX%3A32016L2102

[60] Tsvyatkova D Digital contact tracing applications for COVID-19: a citizen-centred evaluation framework. JMIR Mhealth Uhealth. (2021) 10(3):e30691. 10.2196/30691 PMC8919989 35084338

[61] WC3. Web content accessibility guidelines (WCAG) 2.1 (2018). Available at: https://www.w3.org/TR/WCAG21/

[62] Canada Health Infoway. A framework and toolkit for managing eHealth change (2011).

[63] Canada Health Infoway. A framework and toolkit for managing eHealth change (2011). Available at: https://bpgordersettoolkit.rnao.ca/sites/default/files/CHI_ChangeMgmtGuide_ENG.pdf

[64] Ferlie EB Shortell SM. Improving the quality of health care in the United Kingdom and the United States: a framework for change. Milbank Q. (2001) 79(2):281–315. 10.1111/1468-0009.00206 11439467 PMC2751188

[65] MacMahon ST Alfano M Lenzitti B Lo Bosco G McCaffery F Taibi D Improving communication in risk management of health information technology systems by means of medical text simplification. 2019 IEEE symposium on computers and communications (ISCC) (2019). p. 1135–40

[66] Timmermans S Berg M. The gold standard: The challenge of evidence-based medicine. Philadelphia, PA: Temple University Press (2003).

[67] Carroll N Richardson I. Mapping a careflow network to assess the connectedness of connected health. Health Informatics J. (2019) 25(1):106–25. 10.1177/1460458217702943 28438102

[68] Yu KH Beam AL Kohane IS. Artificial intelligence in healthcare. Nat Biomed Eng. (2018) 2(10):719–31. 10.1038/s41551-018-0305-z 31015651

[69] Yang R Shin E Newman MW Ackerman MS. When fitness trackers don’t “fit”: end-user difficulties in the assessment of personal tracking device accuracy. Ubicomp 2015 - proceedings of the 2015 ACM international joint conference on pervasive and ubiquitous computing (2015). p. 623–34

[70] ISO. Standards by ISO/IEC JTC1/SC 42 - artificial intelligence, iso.org (2023). Available at: https://www.iso.org/committee/6794475/x/catalogue/ (Accessed August 25, 2023).

